# Contested notions of challenges affecting Community Health Workers in low- and middle-income countries informed by the Silences Framework

**DOI:** 10.1186/s12960-021-00701-0

**Published:** 2022-01-06

**Authors:** David Musoke, Mathew Nyashanu, Henry Bugembe, Grace Biyinzika Lubega, James O’Donovan, Abdullah Ali Halage, Linda Gibson

**Affiliations:** 1 Department of Disease Control and Environmental Health, School of Public Health, College of Health Sciences, Makerere University, Kampala, Uganda; 2 School of Social Sciences, Nottingham Trent University, Nottingham, UK; 3 Community Health Worker, Wakiso District, Uganda; 4 Department of Education, University of Oxford, Oxford, UK

**Keywords:** Community Health Workers, Challenges, Silences Framework, Religion, Culture, Patriarchy, Suspicion, Gender, Workload

## Abstract

Despite increasing evidence of the challenges affecting Community Health Workers (CHWs) such as those related to training, supportive supervision and remuneration, there is a need to explore concerns and challenges from the perspective of CHWs themselves. This commentary highlights some of the contested and unexplored notions of challenges affecting CHWs in low- and middle-income countries (LMICs) informed by the Silences Framework. This framework defines experiences that are under-explored, misunderstood or difficult to share because of the often invisible power relations within communities, but also in setting the research agenda. These challenges include the heavy workload imposed by several stakeholders, dealing with religious and cultural practices, and gendered barriers of care. The workload of CHWs is a major source of stress and anxiety as they have to balance both government and other stakeholders’ agendas to deliver interventions with their own need to provide for their families for those whose work is unpaid. The tensions of CHWs carrying out their work among members of the community whose religious or cultural beliefs are different from theirs also needs to be considered. Gender issues are an impediment to the work of CHWs, particularly with community members of the opposite sex around sensitive health issues. Lastly, CHWs have found themselves victims of domestic suspicion while fulfilling their duties in communities, such as when seen having conversations with spouses of other individuals in the community. Solutions to these challenges need to be co-produced with CHWs to both to strengthen their relationship with the communities they serve and shape more sustainable interventions for delivery of healthcare in LMICs.

## Introduction

In 2006, the World Health Organization (WHO) highlighted the lack of skilled human resources for health as a major barrier to achieving Universal Health Coverage (UHC) [1]. As a result, the World Health Report then proposed task-shifting strategies as a potential solution to deliver new interventions and basic curative and preventive care, especially in low-income countries. This strategy includes the use of Community Health Workers (CHWs) to deliver primary health services in their communities, particularly in low- and middle-income countries (LMICs). ‘Community Health Worker’ is an umbrella term, broadly used to describe lay people who live and work closely with local communities, provide basic health care services, and have the potential to act as change agents [2–4]. They can be men or women, literate or illiterate, young or old, and paid or unpaid depending on the country or context they work in [2]. The specific roles and activities assigned to CHWs vary across LMICs [5]. Historically, CHWs have predominantly been involved in the provision of primary health care (PHC), with a strong emphasis placed on health promotion, disease prevention, collection of health data, and management of maternal and child health challenges [6]. Specific activities performed by CHWs are comprehensive and include home visits, promotion of safe water and sanitation, first aid, treatment of simple and common ailments, health education, nutrition promotion, disease surveillance, supporting maternal and child health, enhancing family planning, communicable and non-communicable disease control, community development, referral of patients, record-keeping, and collection of data on vital events [7]. An increased focus has been placed on the role of CHWs in supporting and strengthening health systems in recent years, yet challenges affecting their work are yet to be fully explored and addressed. If we consider them as major stakeholders in attaining the Sustainable Development Goals (SDGs), particularly SDG 3 of “Ensuring healthy lives and promoting well-being for all at all ages”, it is important to understand the challenges affecting them. However, the stakeholders that have majorly been involved in making decisions concerning CHWs include policy-makers, academics, and non-governmental organisations (NGOs) working with them. Although evidence exists regarding the challenges affecting CHWs including those related to training, supportive supervision, remuneration, data collection, availability of supplies, and community engagement [8–10], there is a pressing need to explore the concerns that have received less attention, specifically from the perspective of CHWs themselves. These concerns include the heavy workload imposed on CHWs by several stakeholders, dealing with religious and cultural practices during the course of their work, and gendered barriers of care in the community. Understanding these challenges provides subsequent opportunities for designing interventions to address them, in order to improve performance of CHWs as they contribute to achieving UHC.

Our interest in CHWs as a recognised workforce comes from over ten years of implementing projects in Wakiso and Mukono districts in Uganda. The focus of most of our interventions have been on enhancing the capacity of CHWs through supporting their training, supervision and motivation [11]. Building on the measured success of our interventions, the projects have been rolled out to other areas using a peer-to-peer approach as a way to build evidence for scale up and sustainability. The CHWs we support have been key contributors to local and national stakeholder events where they have shared their own first-hand experiences of supporting community health. Indeed, they have participated and spoken at several events including the first international CHW symposium held in Kampala [12] during which their voices were heard by various stakeholders including local and global policy-makers. In recent years, the CHWs have developed in confidence in relation to their roles, diligently delivering health care, and grown in status within their communities but also, increasingly, within the health system. Opportunities of hearing CHW voices in the past has given us the opportunity to understand the importance of investing in them as a workforce, but also appreciate the need to have their perspectives inform decisions at local and international levels. This commentary therefore highlights some of the contested and unexplored notions of the challenges affecting CHWs in LMICs informed by the Silences Framework.

## Theoretical framework informing the article

This article is informed by the Silences Framework or concept of ‘Screaming Silences’ [13]. Screaming Silences defines areas of research or experiences that are under-explored, misunderstood, or difficult to share among individuals because of the invisible power dynamics at play in setting health and development agendas. The broader theoretical approaches underpinning this framework include aspects of patriarchy [14], and critical based approaches focusing on the contested notion of eliciting views through the lens of individuals experiencing marginalisation [15]. Indeed, despite their important and vital contributions to health systems strengthening initiatives, CHWs have been marginalised for many years. They are largely unpaid or underpaid, their voices are often unheard, they often lack recognition as part of the health workforce, are not always involved in decision-making processes, and are traditionally positioned at the bottom of the health worker hierarchy [16, 17]. In this article, we explore the challenges faced by CHWs as identified by them in conversation. Historically, the challenges faced by CHWs have been portrayed largely through the perspective of academics, thereby perpetuating the marginalisation and exclusion of the views of CHWs themselves. In addition, despite vast published literature on CHWs, authorship has largely excluded CHWs which raises concerns regarding knowledge production [18].

This framework is based on anti-essentialist viewpoints which assert that there is no one version of reality in a given society, but that reality is constructed and has several dimensions [15, 19]. This approach aligns with our epistemological beliefs, in that we value individual and group interpretations of events and human experiences as a valid contribution to an accepted evidence base. In addition, the approach focuses on studying the effects of power in shaping community realities [19–21]. By being informed by the Silences Framework, which is based on critical perspectives, we are able to bring personal and professional scrutiny to the work of CHWs regarding the challenges they face. In this article, we draw on our vast experience of working and interacting with CHWs in Uganda, as well as our involvement in CHW programming around the world particularly in LMICs for several years. In addition, one of the authors of the paper (HB) is a CHW working in Uganda who provides first-hand perspectives on his role and experiences in a rural setting in the country.

## Challenges affecting CHWs

A significant focus of the role of CHWs is to engage with issues faced by the communities they serve, many of which go beyond their formal capacities [22]. CHWs often work under difficult conditions, with little or no immediate technical assistance owing to poor recognition of their activities by many governments in LMICs [23]. A large amount of research has been conducted documenting the challenges faced in their day-to-day work in communities [8, 9]. However, the contribution and shaping of final research outputs concerning CHWs regarding setting the agenda for community development has largely been dominated by professionals and academics [24]. Whereas the impact of challenges facing CHWs is evident, current solutions to these challenges are heavily driven by the influence of professionals whose work environment drastically differs from that of CHWs. Many of these challenges have been explored predominantly from a technical perspective, with less emphasis placed on highlighting the perspectives and voices of CHWs [25, 26]. Some of the challenges that have impeded the smooth running of the work of CHWs as identified by themselves include, but not limited to, overburdening responsibilities, challenging religious and cultural practices in the communities they serve, and gendered barriers of care. These challenges will be explored in more detail in the following sections.

### The burden of workload and expectations

The way in which CHWs are perceived by members of their communities can be problematic, since they are often viewed as paid government workers who receive a regular salary and appropriate material support to carry out their work-related duties [27] which is often far from reality. The assumption that all CHWs are paid is a commonly held misconception, despite calls from the international community to appropriately remunerate them [28]. For example, in Uganda where the majority of our work is based, CHWs remain unpaid and a voluntary workforce [11]. This expectation gap means that CHWs often face balancing dilemmas of trying to provide high quality and diverse PHC services with relatively limited support [29]. Often, CHWs feel they do not meet the needs of the community due to the unrealistic expectations that are placed upon them [30]. Their efforts to signpost community members to formal health services are often received with ridicule and scorn, as communities view them as full-time government employees and place an expectation on them to always provide a needed service [31]. As a result, they are inundated with multiple responsibilities [32], often in a piece meal fashion, and can often be viewed as a ‘magic-bullet solution’ regarding health concerns in the community. However, given the limited training and supervision they often receive, coupled with other issues such as funding and resource shortages, they have limited capacity in fulfilling the tasks expected of them. This burden of expectation can have dire negative consequences for the CHWs themselves, such as making significant out-of-pocket payments in order to provide PHC services and fulfil the expectations of the community [33].

### Religious and cultural practices in communities

The role of religion and differing cultural practices has been noted to negatively impact the ability of communities to live together in harmony, resulting in disjointed social ties [34]. This can consequently have adverse impacts on how CHWs undertake their work within their communities. For example, some community members with extreme religious beliefs and practices sometimes do not allow CHWs with differing beliefs to visit their households. This barring of CHWs from homes is not only visible in religious practice, but also when the CHWs are from another tribe whose cultural practice differs from members of the community. These tensions are historically rooted and can be deeply embedded within communities [35]. Furthermore, health issues such as HIV, are sometimes considered sensitive—at times taboo—subjects due to religious and cultural beliefs. This has resulted in divided opinions among communities as to how they should be addressed and managed [36]. Indeed, many CHWs have found it difficult to access certain families and freely discuss and give advice on sensitive health issues such as contraception and safe sex. On these occasions, communities have directed their anger towards CHWs and at times blamed them for risky behaviours in their communities such as unprotected sex [37]. This blame attached to CHWs can threaten their health, security and well-being in their community.

### Gendered barriers of care

There is a gap in knowledge around the gendered nature of care in the CHW workforce in LMICs. It is widely recognised that gender inequality in health services workforce means that male and female workers have unequal access to employment and career-advancement opportunities, unequal skills acquisition, and unequal security when delivering services [38]. Care labour, as both a paid and unpaid activity, has traditionally been seen as women’s work which is replicated among CHWs in many LMICs. The patriarchal nature of many cultures in which CHWs work means that men often regard themselves as the custodians of the communities in which they live [39]. In addition, there are strong gendered roles within families all of which act as barriers to the delivery of healthcare. This has proven challenging for female CHWs to complete their work in households where men hold such views [40]. Many female CHWs have suffered embarrassment in front of families for trying to speak to men about the different health issues affecting them such as on reproduction. In other settings, CHWs often have to seek male approval before they are allowed to speak to female members of households. On many occasions CHWs have travelled long distances, only to be refused permission by the male head of household to talk to the family for health promotion including discussing sensitive health issues. This is a stressful experience especially when CHWs have to spend a lot of their time moving between households in the communities [41]. This concern, although common from our first-hand experiences, is often not reflected in literature surrounding challenges faced by CHWs, which instead largely focuses on the technical challenges surrounding their roles such as training, remuneration and supplies.

Suspicion of spouses is one of the main sources of domestic violence in many communities [42]. On many occasions, CHWs have found themselves victims of domestic mistrust while fulfilling their duties in communities. Many male CHWs are apprehensive of holding health promotion conversations with wives of absent spouses, out of fear of being accused of flirting with them. This is also exacerbated when the spouse feels uncomfortable discussing subjects that the CHWs may be talking about, for example if related to sexuality. In the same way, male CHWs have found it hard to talk to families of absent fathers. Female CHWs have also been prevented from discussing certain issues such as sexual health with spouses of women in communities. Many women fear that their spouses will become engaged in extra-marital affairs if topics concerning sex are discussed. This has resulted in female CHWs being told to leave households by female members, leaving them unable to complete their responsibilities.

## Discussion

In line with the Silences Framework, the agenda on issues relating to CHWs have largely been championed and discussed by national and international stakeholders including professionals and academics, with a bias towards their own interest and relevancy to their work [43]. The need to listen more to voices of CHWs and communities is only more recently starting to be recognised [25]. Indeed, giving CHWs opportunities to engage in discussions concerning their work, and actively involving them in planning, delivery and evaluation of their activities would help address existing invisible power dynamics. In addition, there is need for more recognition of the role of CHWs in health systems by stakeholders at community, national and global levels. In this commentary, in which one of the authors is a CHW, we have highlighted some of the key unexplored and contested notions affecting the work of CHWs, drawing on our experience of working in Uganda’s health system and beyond. In addition, we suggest possible pathways that can be used by various stakeholders concerning the work of CHWs such as governments and NGOs to address these challenges (Table 1). Owing to limited resources, many national and international organisations, including governments, are utilising the services of CHWs to facilitate their programmes in communities [5, 44]. The management of CHW programmes is often divested to different organisations across LMICs [45], including NGOs. In doing so, many of these organisations have rarely considered the impact of all their interventions on the capacity of CHWs to be effective, and personal challenges faced by CHWs working with communities. The highest priority is often around training CHWs to undertake their designated work while keeping costs low. While CHWs are strategically positioned to carry out community development work on behalf of governments and other stakeholders, it is important that the burden of their work is adequately assessed and constantly reviewed not to compromise their performance. Overburdening CHWs results in stress and anxiety, leading to lost working hours. Issues of appropriate remuneration and career recognition can be overlooked when professionals and academics set the agenda for development with regard to CHWs. The issue of remuneration and career for CHWs is also part of a wider set of discussions for building strong publicly financed health systems globally which value workers and listen to their experiences and voices [28].

**Table 1:** Contested and unexplored challenges affecting CHWs in low- and middle-income countries

Challenges affecting CHWs	Suggested pathways to address the challenges
Heavy workload and high expectations	Routine assessment of the burden of CHW work Paying attention to remuneration and career recognition of CHWs Giving CHWs opportunities to engage in discussions concerning their work Involving CHWs in planning and evaluation of their activities More recognition of the role of CHWs in health systems
Religious and cultural practices	Enhancing community involvement in the work of CHWs Discussing with CHWs issues concerning religion and culture in the community Supporting CHWs in working among communities with different cultural and religious beliefs
Gendered barriers of care	Using a multi-dimensional approach while supporting the work of CHWs in communities Understanding the dynamics of male and female influence in communities Conducting more research on patriarchy and its impact on the performance of CHWs

Culture and religion are central to how communities interact and communicate with each other [46]. Professionals have always considered culture and religion as a barrier to accessing lines of communication and acceptability of issues in health promotion [47]. These issues have to be dealt with at community level to facilitate stakeholders’ work, but usually marginalise the experience of CHWs when, for example, they are promoting sensitive topics in their communities. To help address this challenge, we call for greater attention to be paid to understanding challenges at individual and community levels. We could begin by asking what personal threats CHWs are likely to experience in a different culture whose practice is opposed to the health communication messages they are trying to convey. Similarly, it would be important to explore how CHWs can be supported in delivering health promotion and education to diverse communities with different cultural and religious beliefs. In light of this, the voices of CHWs should be listened to by various stakeholders and appropriate action taken. This is important because a sense of safety and confidence are central to CHWs realising their goals when working with communities [48].

The impact of gender and patriarchy on health and well-being of communities is not a new phenomenon. The concept has appeared in some of the earliest writing on community and family health [49]. The usual focus in confronting patriarchy has been to address its impact on the health and well-being of women [50]. However, little has been done to assess its impact on how CHWs undertake their work in communities. Indeed, patriarchy is an impediment to the work of both male and female CHWs. For example, many male CHWs have found it difficult to have conversations with women around sensitive health issues such as sexual health in the absence of their spouses. In some circumstances, male CHWs have found themselves in trouble after being found by other men talking to their spouses about sensitive issues without approval. This slant of patriarch among CHWs has not been documented enough in literature. Therefore, the concept of patriarchy and its impact as a barrier for the work of CHWs needs further exploration. Patriarchy, as a gendered set of power relations, is embedded in the structures of culture, religion and society. As we have discussed how patriarchy impacts on the work of CHWs, there is need for its deeper analysis in the health and development field. Although efforts to address patriarchy are likely to be complex and challenging [51], use of a multi-dimensional approach can also help in easing the difficulties experienced by both organisations and CHWs when carrying out community work. Indeed, understanding the dynamics of the male and female influence in communities as well as involving opinion leaders can be a good starting point [52].

## Conclusion

Many of the concerns affecting CHWs that exist in literature are viewed through a technical lens, and thus neglect many of the other concerns and challenges faced by CHWs. However, challenges related to the high workload, religion, culture and gender as experienced by CHWs need to be addressed as a priority. Interventions to address these challenges could provide a safer working environment for both CHWs and communities. For governments and other stakeholders, listening to and valuing CHW voices and experiences can help shape more sustainable solutions to the complex health needs of their communities. We argue that investing in this workforce as representative of local communities gives national and global governance structures the opportunity to align to understanding health as a social rather than technical process, and to build fairer health systems based on UHC principles shaped by communities.

## Data Availability

Not applicable.

## Abbreviations

CHWs: Community Health Workers
LMIC: Low- and middle-income country
NGO: Non-governmental organisation
PHC: Primary Health Care
SDGs: Sustainable Development Goals
UHC: Universal Health Coverage
WHO: World Health Organization
